# Development of apical out trophoblast stem cell derived organoids to model early human pregnancy

**DOI:** 10.1016/j.isci.2025.112099

**Published:** 2025-02-25

**Authors:** J. Zhou, M.A. Sheridan, Y. Tian, K.J. Dahlgren, M. Messler, T. Peng, A. Zhao, T. Ezashi, L.C. Schulz, B.D. Ulery, R.M. Roberts, D.J. Schust

**Affiliations:** 1 Duke Obstetrics & Gynecology, Duke University School of Medicine, Durham, NC 27710, USA; 2 Department of Obstetrics, Gynecology, and Women’s Health, School of Medicine, 1 Hospital Dr, University of Missouri, Columbia, MO 65212, USA; 3 Bond Life Science Center, University of Missouri, 1201 Rollins St, Columbia, MO 65211, USA; 4 College of Engineering, University of Missouri, Lafferre Hall, W1024, Columbia, MO 65211, USA; 5 Department of Histology and Embryology, School of Basic Medcine, Tongji Medical College, Huazhong University of Science and Technology, Wuhan 430030, P.R. China; 6 Colorado Center for Reproductive Medicine, 10290 RidgeGate Circle, Lone Tree, CO 80124, USA; 7 Department of Biochemistry, University of Missouri, 503 S College Ave, Columbia, MO 65211, USA; 8 Division of Animal Sciences, University of Missouri, Columbia, MO 65211, USA

**Keywords:** Physiology, Molecular biology, Cell biology

## Abstract

The development of trophoblast organoids has enabled investigation of placental physiology, disease, and early maternal-fetal interactions during a previously restricted stage of pregnancy. A key shortcoming in existing trophoblast organoid methodologies is the non-physiologic position of the syncytiotrophoblast (STB) within the inner portion of the organoid, which neither recapitulates *in vivo* placental villous morphology nor allows for facile modeling of STB exposure to the endometrium or the contents of the intervillous space. Here, we have successfully established apical-out human trophoblast stem cells (hTSC)-sourced organoids with STB forming on the surface of the organoid. These organoids can also be induced to give rise to the extravillous trophoblast (EVT) lineage, which invades into an extracellular matrix-based hydrogel. Compared to previous methods, our organoids more closely mimic developing human placental architecture, offering a novel platform to study normal and abnormal placental development and to model exposures to pharmaceuticals, pathogens, and environmental factors.

## Highlights

• We developed an apical-out trophoblast organoid model with surface STB and internal CTB
• This model mimics *in vivo* villous placental structure and can be differentiated into EVT
• Our model optimizes physiological relevance for studying placental development and disease
• This system enables investigation of environmental exposures and maternal-fetal interactions

## Graphical abstract

**Figure:**
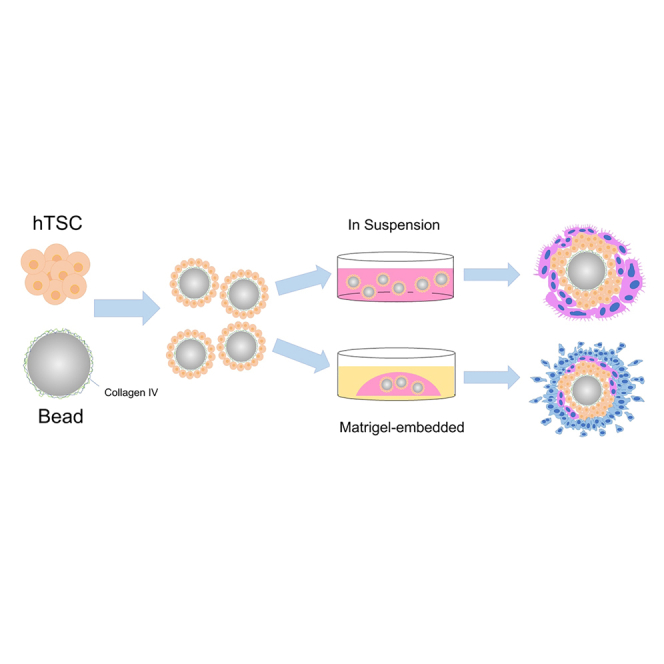

## Introduction

Trophoblast is a specialized, conceptus-derived cell type of the human placenta. All of the trophoblast lineages are generally thought to arise from the blastocyst trophectoderm (TE),^1^^,^^2^^,^^3^ and their coordinated proliferation and differentiation is essential for a successful pregnancy.^4^ Initially, some of the trophectoderm cells merge to form a leading edge pre-villous syncytial mass that implants into the maternal endometrium. Subsequently, the three major trophoblast subpopulations of the villous stage placenta are established and maintained throughout pregnancy: cytotrophoblast cells (CTB), extravillous trophoblast cells (EVT), and syncytiotrophoblast (STB), although there may be multiple functional sub-types within these larger groupings.^5^ The underlying CTB are a proliferative population that can give rise to either the STB lineage that lines the outer surface of the villi, or the EVT lineage at the point where some villous tips anchor to the maternal decidua (anchoring villi).^6^^,^^7^^,^^8^^,^^9^ Impaired trophoblast development and function are thought to lead to pregnancy complications, including miscarriage, preeclampsia, and intrauterine growth restriction.^10^^,^^11^^,^^12^

Efforts to understand the human placenta have been plagued by a lack of functional experimental models.^13^ Our current understanding of human placental morphology during early pregnancy relies upon rare, archived samples and non-human primate models. As a consequence, fundamental questions about the initial stages of human trophoblast invasion and placentation remain unanswered. Historically, trophoblast isolated from human placentas have proven to be short-lived in culture and to rapidly differentiate.^14^ This limitation was recently resolved by the isolation and derivation of trophoblast stem cells^15^ and the development of trophoblast organoids.^16^

Several groups have reported using stem cells or first-trimester placental cells to establish three-dimensional (3D) placental organoids that contain both STB and EVT.^17^^,^^18^^,^^19^^,^^20^ While these 3D organoids are thought to provide a functional model of the human placenta, their polarity is inverted. This “inside-out” morphology, which is likely a consequence of the Matrigel droplet in which they are embedded serving as a basement membrane, results in an outer layer of CTB cells and formation of multi-nucleated STB within the center of the organoid. The goal of the present work has been to develop a method to invert this polarity, creating human trophoblast organoids from trophoblast stem cells that more closely resemble the *in vivo* structural organization of the human placenta.

## Results

### Generation of apical-out STB trophoblast organoids from hTSC that are characterized by an outer layer of STB

**Figure 1:**
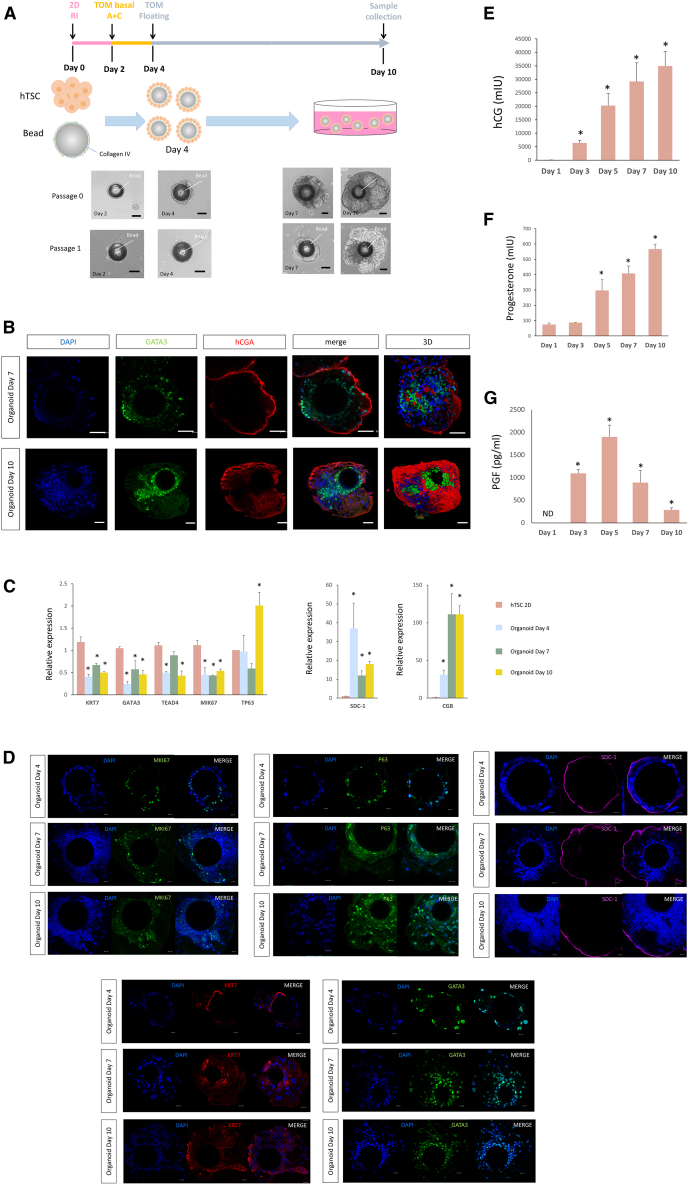
Establishment of apical-out trophoblast organoid cultures from hTSC cells (A) The hTSC cell line (CT27) was maintained under traditional 2D-culture conditions. To develop our organoid cultures, these cells were dispersed and incubated with collagen IV-coated beads in TSC medium supplemented with 5 μM Y27623 for 2 days (pink line). The medium was then switched to TOM basal medium containing A83-01(500 nM) and CHIR (1.5 μM) (TOM basal A + C) for an additional 2 days (yellow line). The developing organoids were then treated with complete TOM medium until day 10 (gray line). Bright-field images of hTSC-derived trophoblast organoids from passage 0 and passage 1 are depicted. Scale bars, 200μm. (B) Immunostaining of hCGA and GATA3 in STB organoids on Day 7 and Day 10. hCGA is expressed in multinucleated areas lining the outer surface of the organoids while the mononucleated cells closer to the center of the organoids are GATA3 positive. Reconstructed 3D confocal images showing the distribution of hCGA and GATA3. Scale bars, 50μm. (C) Quantitative real-time PCR analysis of CTB markers (upper panel) and STB markers (lower panel) in hTSC (2D culture) and STB organoids on Day 4, Day 7, and Day 10. KRT7, GATA3, TEAD4, and MKI67 transcript levels were higher in hTSC than in organoids, while hCGB and SDC1 transcript levels were much lower in hTSC than in organoids. Data were normalized to GAPDH expression. Presented values are the means ± SDs for three separate experiments Presented values are the means ± SDs for three separate experiments. Data followed over time were subjected to two-way ANOVA, followed by the Bonferroni test for pairwise comparisons. ∗*p* < 0.05. (D) Immunostaining for MKI67, GATA3, KRT7, P63, and SDC-1 in trophoblast organoids on Day 4, Day 7 and Day 10. Trophoblast cells in the center of the organoids express the CTB markers GATA3 and KRT7, MKI67 staining labeled proliferating trophoblast cell, and the STB marker SDC-1 is detected on the surface of the trophoblast organoids. Scale bars, 50μm. (E–G) Images are representative of three biological replicates. Daily production of three placental hormones hCG (E), progesterone (F), and PGF (G) was assessed in the culture medium by ELISA over the time course of organoid culture. The medium was replaced on day 2, day 4, day 6, and day 9 of culture, 24h before collection. Presented values are the means ± SDs for three separate experiments. Data followed over time were subjected to two-way ANOVA, followed by the Bonferroni test for pairwise comparisons (*n* = 3). ∗*p* < 0.05.

CT27 hTSCs^15^ (1st trimester placenta derived, female) maintained in 2D culture were dispersed and cultured with collagen IV-coated polystyrene beads of 200 μm diameter for 2 days. During this initial culture period, all functionalized beads became ensconced by hTSCs. The bead-associated trophoblast organoids were expanded in trophoblast organoid medium (TOM)^20^ in the absence of forskolin to induce syncytialization, with organoids reaching an average size of 600 μm by day 10 (Figure 1A). These organoids could be passaged to regenerate their structures within 7–10 days (Figure 1A). To evaluate the morphology of the trophoblast organoids after 10 days in culture, they were fixed, paraffin-embedded, sectioned, and stained with hematoxylin and eosin (H&E). The trophoblast organoids growing around a bead were composed of multiple (3–4) inner layers of mononuclear cells surrounded by a semi-continuous multinucleated layer (Figures S1A, left panel and S1B), recapitulating the organization of placental villi *in vivo*. These bead-associated, apical-out organoids contained numerous vacuoles, also typical of the STB *in vivo*^21^^,^^22^^,^^23^ and during organ culture.^24^^,^^25^ Cell aggregates without beads exhibit a random distribution of multinucleated cells, often internal, and an increase in vacuolar structures, indicating globally robust cell fusion (Figure S1A, right panel). We have replicated our apical-out methods using CT29^15^ (1st trimester placenta derived, male), BTS5^15^ (blastocyst derived, female) and BTS11^15^ (blastocyst derived, male), with resulting generation of organoids that also demonstrate a apical-out morphology (Figure S1C).

To further characterize our apical-out trophoblast organoids, quantitative PCR (qPCR) and immunofluorescent staining was performed on organoids collected on culture days 4, 7 and 10. Using qPCR, we detected significantly higher levels of SDC-1 (Syndecan 1) and CGB (chorionic gonadotropin beta) transcripts in trophoblast organoids than in undifferentiated hTSCs grown in a two-dimensional configuration in tissue culture wells (Figure 1C). Expression of the STB markers, SDC-1, CGB and CGA, was detected mainly in multinucleated patches near the outside surface of the organoids, while antigens such as MKI67 (marker of proliferation Ki-67), GATA3 (GATA binding protein 3), p63 (tumor protein p63) and KRT7 (cytokeratin-7), were largely concentrated in the mononucleated cells inside the organoids (Figures 1B, 1D, and S1F). CT27-derived organoids embedded in Matrigel and cultured following an established protocol were employed as a comparative control. However, distinct markers of STB, including CGA, CGB, and SDC-1, were expressed within the central areas of these organoids, while the outer layers expressed the CTB marker, GATA3 (Figure S1E).

The apical-out trophoblast organoids secrete hCG into the medium, which is detectable after 1 day in culture and gradually increases until day 10 (Figure 1E). Like hCG, progesterone (P4) release from bead-associated, apical-out organoids also increased over time (Figure 1F). Production of placental growth factor (PGF) peaked at day 5 in culture and dropped significantly by day 10 (Figure 1G). In order to conduct a comprehensive comparison of the hormone production capacity of apical-out trophoblast organoids vs. Matrigel-embedded organoids, we standardized the levels of hCG and progesterone on Day 10 by normalizing to DNA content. Our findings revealed that apical-out trophoblast organoids exhibited lower secretion of hCG and progesterone when compared to Matrigel-embedded organoids (Figure S1D).

### Ultrastructure of apical-out trophoblast organoids

**Figure 2:**
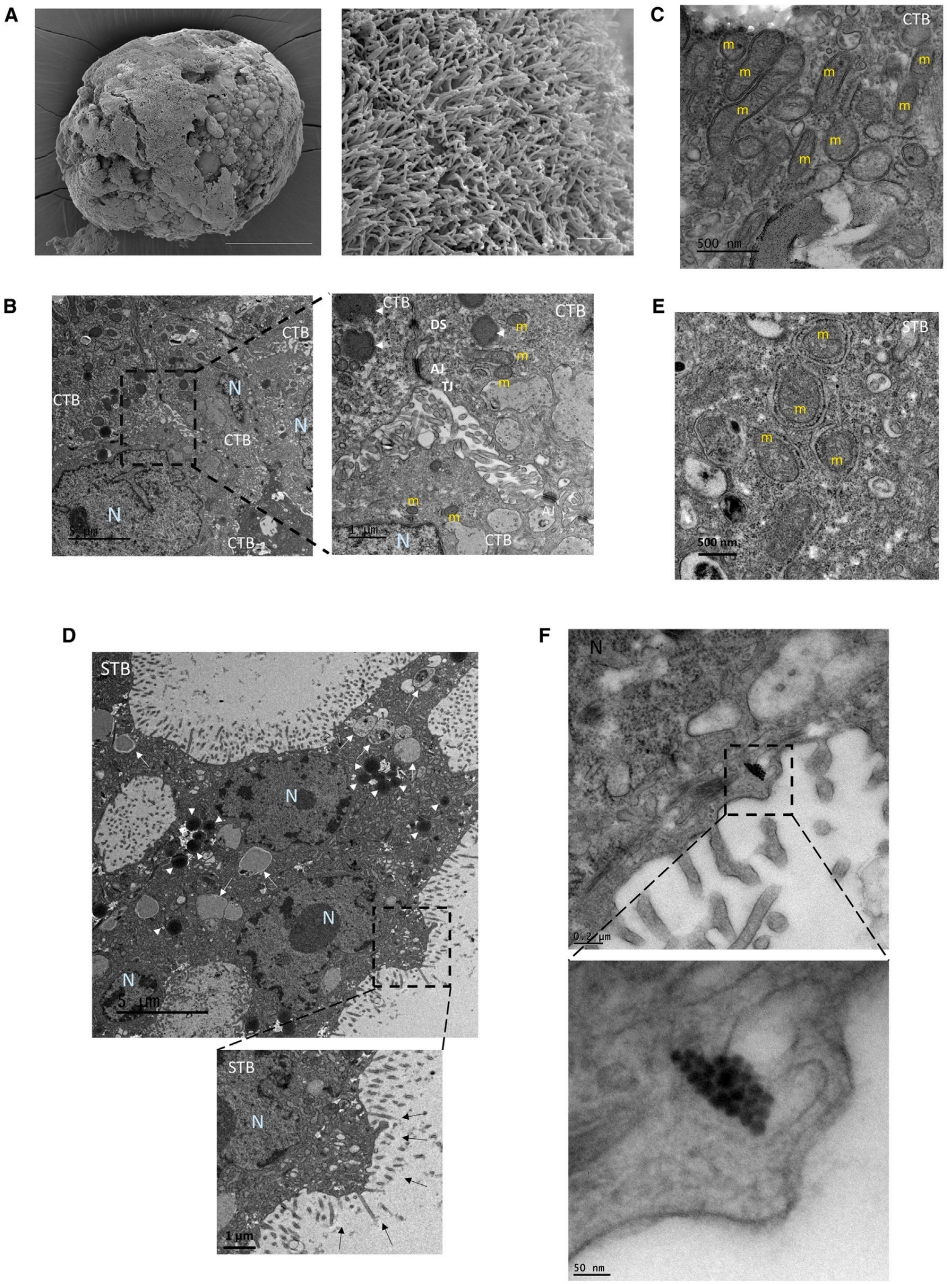
Ultrastructure of CTB and STB in trophoblast organoids (A) Scanning electron microscopy (SEM) images of STB organoids reveals a topography (left panel, scale bar, 100 μm) covered by dense microvilli (right panel, scale bar, 3 μm). (B) Transmission electron microscopy (TEM) images of an “apical junctional complex” between mononuclear CTB from 3D floating STB-covered organoids. Depicted are the tight junctions (TJ), directly beneath the microvilli; adherens junctions (AJ), and desmosomes (DS) below the apical junctional complex. Scale bars, 5 μm and 1 μm. N, Nucleus; m, mitochondria; lysosome (white arrow heads). (C) The larger mitochondria in presumed mononuclear CTB have lamellar cristae. Scale bars, 500 nm. (D) Microvilli (black arrow), lysosomes (white arrow heads) and autolysosomes (white arrow) that can be seen in multinuclear STB at Day 10. Scale bars, 5 μm and 1 μm. (E) STB mitochondria have a dense matrix and vesicular cristae. Scale bar, 500 nm. (F) A nematosome can be seen in a mononuclear trophoblast cell. Scale bars, 200 nm, 50 nm.

Scanning electron microscopy (SEM) and transmission electron microscopy (TEM) were used to visualize common structural features of trophoblast organoids and *in vivo* STB.^20^ Trophoblast organoids contained multinucleated areas surrounded by a continuous cell border and displayed large areas of surface microvilli consistent with normal villous STB structure *in vivo* (Figures 2A and 2D). The internal mononucleated cells in the organoids displayed irregularly shaped nuclei, with chromatin aggregation beneath the nuclear membrane. The plasma membranes of the multinucleated cells tended to be irregular with microvilli projections (Figures 2B and 2D). Mononuclear cells maintained contact with each other through adhesion complexes, including tight junctions, adherens junctions, and desmosomes (Figure 2B). We also noted that the mitochondria found in multinuclear patches (STB) display a distinctive appearance. Similar to prior reports on placental biopsies,^26^ the mitochondria in the multinuclear patches were numerous, round, electron-dense, and contained visible tubular cristae; they were also smaller than those found in the mononuclear cells inside the same organoids (Figures 2C and 2E). The reductions in mitochondrial size and structural alterations in mitochondrial cristae are consistent with the decrease in glycolytic activity and the expansion of steroidogenic activity typically seen in STB *in vivo*.^27^^,^^28^ Also, as previously reported,^29^ numerous autolysosomes and autophagosomes were detected in both organoid cell types (STB and CTB) (Figures 2B and 2D), suggesting a potential to activate autophagy, which is involved in trophoblast fusion.^30^ We also located a non-membrane-bound cytoplasmic organelle, termed a nematosome, in the cytoplasm of a cell within a trophoblast organoid (Figure 2F). Nematosomes have previously been reported in human trophoblast, but their function remains unclear.^31^

### HLA-G+ CGB+ double-positive cells were detected in apical-out trophoblast organoids

**Figure 3:**
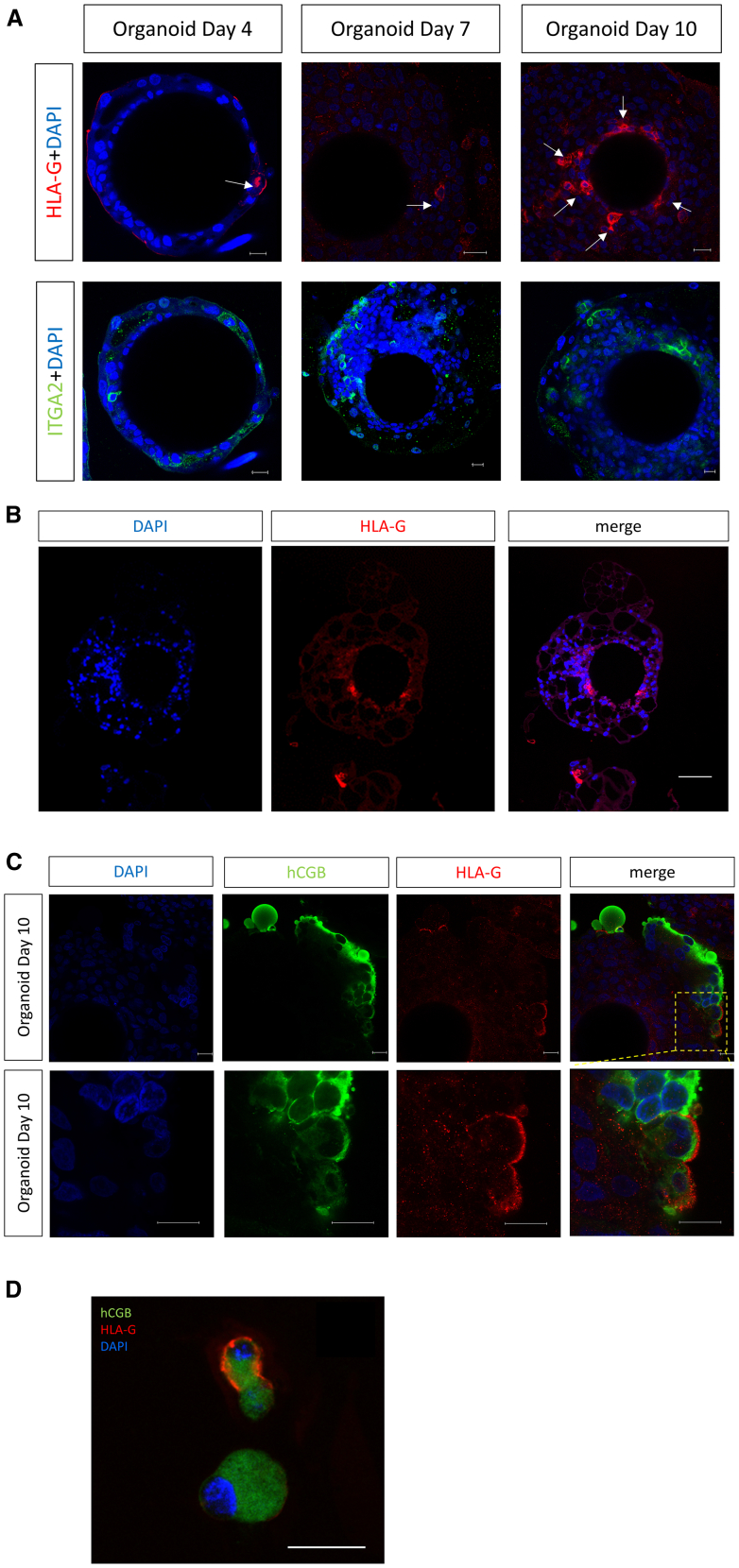
Expression of HLA-G in trophoblast organoids (A) Whole mount staining shows sporadic expression of the EVT markers, HLA-G and ITGA2, in floating STB organoids on Day 4, Day 7 and Day 10. Scale bars, 20 μm. (*n* = 3) (B) Sections of paraffin-embedded STB organoids at Day 10 were immunostained for HLA-G. A small number of cells close to the beads were HLA-G positive. Scale bars, 100 μm. (*n* = 2) (C) Co-staining of HLA-G and hCGB using whole-mounted floating STB organoids with images showing HLA-G staining on hCGB positive cells. Scale bars, 20 μm. (*n* = 3) (D) Some cells shed into the culture medium co-expressed HLA-G and hCGB. Scale bars, 20 μm. (*n* = 2).

It has been reported that a few HLA-G^+^ cells can be found in inverted human trophoblast organoids maintained in a standard proliferation medium, trophoblast organoid medium (TOM).^20^ We also observed sporadic cells in the inner layer of our trophoblast organoids that expressed the EVT marker, HLA-G, on Days 4, 7, and 10 (Figures 3A and 3B). In addition, we occasionally found HLA-G^+^ CGB^+^ SDC-1^+^ cells located on the surface of the apical-out floating organoids; these individual cells could also be retrieved from collected culture media (Figures 3C and 3D).

### Generation of migratory and invasive EVT-like cells from apical-out trophoblast organoids

**Figure 4:**
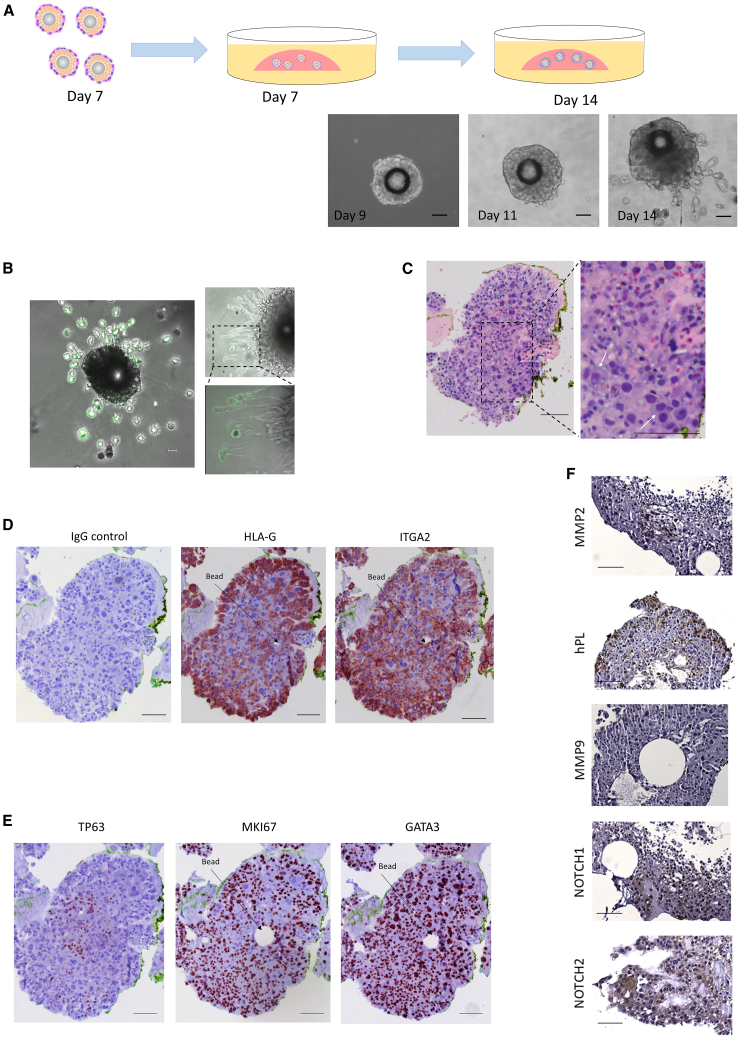
Generation of migratory and invasive HLA-G+ EVT-like cells from trophoblast organoids (A) STB organoids on Day 7 were plated into Matrigel in EVT differentiation medium (EVTM). Phase-contrast images on Day 9, Day 11, and Day 14 (2 days, 4 days and 7 days after placement into the Matrigel drops, respectively). Scale bars, 200μm. (B) Live cell imaging showing cells growing out from organoids in Matrigel and EVTM stained with the anti-HLA-G monoclonal antibody G233. Cells stream out of organoids in two ways: migrating into the Matrigel or adhering to and moving along the plastic dish. Scale bars, 200 μm, 50 μm. (C) H&E staining displayed large cells (compared with the adjacent trophoblast cells) containing two nuclei enclosed in a voluminous cytoplasm (white arrows). Scale bars, 100 μm. (D–F) Day 14 paraffin-embedded sections of CT27 organoids grown in EVTM for 7 days, stained for the EVT markers, HLA-G and ITGA2 (D); the CTB markers TP63, MKI67, and GATA3 (E), the infiltrating trophoblastic markers, MMP2, MMP9, hPL and the column trophoblastic markers, NOTCH1 and NOTCH2 (F). Scale bars, 100 μm.

Two-dimensional cultures of hTSC and human trophoblast organoids can be coaxed to give rise to EVT-like cells by using specialized media (extravillous trophoblast medium; EVTM) that contains a TGFβ inhibitor and neuregulin 1 (NRG1).^15^^,^^20^^,^^32^ We adapted this EVTM protocol for our trophoblast organoids to induce similar differentiation. After 7 days of floating culture in suspension medium (Figure 1A), we transferred the trophoblast organoids into Matrigel droplets and switched to culture in EVTM (Figure 4A). Cells were observed to have migrated outside of the organoid into the Matrigel within 7 days, forming spindle-like structures with typical EVT morphology, as reported by others^33^^,^^34^^,^^35^) (Figures 4A, 4B, S2A, and S2B). H&E-stained sections of the organoids grown in Matrigel and EVTM showed heterogeneous cell populations (Figure 4C). Mononuclear cells containing nuclei of various size and multinucleated patches enclosed by large areas of cytoplasm could be identified. Many of the cells in these organoids at day 14 (7 days in Matrigel) expressed both the CTB column marker ITGA2 (Integrin α2) and the EVT marker, HLA-G,^36^ with the most intense HLA-G staining located on the outermost layer of the organoid (Figures 4D and S2A). ITGA2 has been shown to be expressed in the progenitor cells that comprise a subset of column CTB at the base of anchoring villi in the first trimester and in cells inside “apical-in” organoids made from TS or primary cells, where it is lost upon full differentiation to HLAG+ EVT.^16^^,^^36^^,^^37^ In first trimester placenta, about 70% of ITGA2+ cells are also HLAG+, while the remaining cells express the CTB marker EGFR.^36^ Double-positive cells have also been reported in single-cell RNA-seq analyses.^36^^,^^38^^,^^39^ Whether the ITGA2+, HLAG+ trophoblast progenitor cells are fully committed to the EVT lineage, or also can give rise to villous CTB is unknown.

We identified proliferating cells by staining for MKI67, which is expressed during all cell cycle stages except G0.^40^^,^^41^ Most MKI67+ cells within the organoids cultured in EVTM for 7 days were mononuclear and contained nuclei of consistent size; these were distributed throughout the organoid (Figures 4E and S2D). Another proliferative CTB cell marker, TP63, exhibited limited expression in organoids differentiated under EVT conditions (Figures 4E and S2D). Note that at the time when the trophoblast organoids were initially seeded into the Matrigel in EVTM, they were already surrounded by multinucleated STB and only rare cells expressed ITGA2 (Figure 3A). After 7 days in EVTM, ITGA2 was present in a majority of the mononucleated cells, suggesting that the structures mimicking *in vivo* CTB columns formed and developed in response to Matrigel and EVTM conditions (Figures 4C, S2A, and S2C). Moreover, the presence of hPL (human placental lactogen), notch receptors such as NOTCH1 (Neurogenic locus notch homolog protein 1) and NOTCH2 (Neurogenic locus notch homolog protein 2), in conjunction with the gelatinase, MMP-2, (matrix metalloproteinase-2) was detectable, although MMP-9 was absent in these organoids (Figures 4F and S2E).

## Discussion

Despite its critical role in pregnancy, the placenta is the least understood human organ,^42^ largely as a consequence of practical, legal, and ethical restrictions on studying its development. Recently, several groups have reported the development of trophoblast organoids derived from first-trimester placental tissues, term placental tissues, hTSC, and naive human pluripotent stem cells (hPSC).^17^^,^^20^^,^^43^^,^^44^^,^^45^ Trophoblast organoids (TO) are self-renewing, three dimensional cellular structures generated *in vitro* that recapitulate multiple aspects of placenta development and function.^17^^,^^20^ However, almost all the reported trophoblast organoid models to date, and all of those derived from human TSCs, poorly recapitulate the *in vivo* anatomy of the developing primitive and villous human placenta. Both of these placental structures have their maternal-facing surfaces covered by a layer of multinucleated syncytialized trophoblast. This STB layer has several functions, including serving as: (1) the major transporting epithelium in the placenta, with capacity for polarized gas and nutrient transfer,^46^ (2) a physical and immunologically active barrier to fetal infection and toxin exposures, and (3) an induction site for immunotolerance of the antigenically foreign fetus in normal pregnancies^47^^,^^48^ through the shedding syncytial nuclear aggregates and exosomes that interact with the maternal immune system. Here, we present an improved trophoblast organoid model derived from hTSC in the absence of forskolin exposure that presents its multinuclear layer at the outer surface of the organoids, more closely mimicking the *in vivo* architecture of the implanting embryo and villous placenta than prior models.

The human blastocyst is a roughly spherical structure with a diameter of about 200 μm at approximately 7 days post fertilization^49^ when it undergoes implantation.^50^ Size equivalent beads were modified via amidation to offer opportunities for covalent coupling with collagen type IV.^51^ This collagen can provide structural support to regulate adhesion, migration and survival of cells,^52^ and has been reported to affect the invasive behavior of trophoblast cells at the fetal-maternal interface.^53^ hTSCs were exposed to amidated, collagen IV-coated beads and then cultured in suspension medium. By day 4, the developing organoids are multilayered with a syncytialized outer surface layer beginning to form and an inner core of proliferative single CTB-like cells that are capable of further differentiation (Figures 1B and 1D). By day 7–10 of culture, in addition to the formation of multiple cell layers on the bead surface, a layer of cells with ultrastructural characteristics of intermediate-stage trophoblast, as described by Jones and Fox,^54^ can be detected between the undifferentiated CTB cells near the bead and the outer syncytium (Figure 2B).^55^^,^^56^^,^^57^^,^^58^ The spatial arrangement of growth and differentiation around the bead occurs in all directions, contrasts with the flat structure of conventional 2D cultures and promotes cellular interactions and differentiation analogous to *in vivo* tissue environments.

The human placenta is an endocrine organ, generating a wide array of hormones required for the establishment and maintenance of pregnancy. We explored the secretory activity of apical-out trophoblast organoids. Typical of STB *in vivo*, the apical-out trophoblast organoids secrete hCG, P4 and PGF into the surrounding culture media, although secretion of the latter occurs over a limited time and does not occur until a later time point in the culture period. Although delayed onset of robust secretion is also characteristic human pregnancy,^21^ it remains uncertain why PGF secretion exhibits this overall pattern in our organoid cultures (Figure 1G). One possible explanation is the absence of maternal stimuli such as low O_2_ concentrations as observed in the uterus, inflammatory cytokines, growth factors and hormones normally present during implantation and placental development *in vivo.*^22^^,^^23^^,^^24^^,^^25^ When comparing 2D hTSC-derived inverted organoids cultivated under the conditions outlined by Turco et al.,^20^ with our apical-out trophoblast organoids, we observed reduced production of hCG and progesterone. Specifically, for hCG production, CT27 and BTS11 decreased to 53% and 32% respectively. In the case of progesterone, these levels decreased to 63% and 30%, respectively. Our apical-out STB organoids are grown in the absence of Matrigel and this difference in hormone secretion may be related to the presence of this particular tumor-derived substrate, which could promote non-physiologic cell growth, aggregation and associated secretory function, as has been reported in other systems.^59^^,^^60^ It remains unknown which levels of hCG and progesterone truly represent those found *in vivo* at the maternal-fetal interface during early placentation, particularly around the time of implantation. Matrigel, being a crude extract composed of a wide variety of proteins, including growth factors, functions as a reservoir for these factors and influences both cultured cells and their microenvironment.^61^ Matrigel’s susceptibility to batch variations poses challenges for data reproducibility. Further, Matrigel can make co-culture conditions for heterologous cell types difficult to optimize. Our ultimate goal is to culture 3D trophoblast organoids in the presence of such heterologous cells, including epithelial and stromal endometrial cells and decidual immune cells.

The STB of the human placental barrier is covered with microvilli that are exposed to the maternal blood in the intervillous space.^62^ This microvillous surface is vital for placental nutrient exchange, transfer of oxygen, immune protection, excretion of waste products and secretion of hormones.^63^^,^^64^ Microvilli are also involved in the first intercellular interactions between mother and embryo during implantation, maintaining close adhesion between the trophoblast surrounding the implanting blastocyst and the uterine epithelium.^65^ The syncytialized outer layer of our trophoblast organoids exhibit well-organized apical microvilli characteristic of the STB surface *in vivo*^66^^,^^67^ (Figure 2A). The sporadic HLA-G^+^ CGB^+^ SDC-1^+^ cells on the surface of our organoids or retrieved from collected trophoblast organoid culture media (Figures 3C and 3D) may perhaps model the remarkable and unique, pre-villous, invasive primitive STB. Primitive STB secretes massive amounts of hCG to rescue the maternal corpus luteum and thereby maintain pregnancy, but also migrates into the maternal decidua to establish initial interactions with the decidual stromal cells and glands as well as decidual immune cells, including maternal dNKs.^68^^,^^69^ This hypothesis will be investigated in future experiments but is consistent with data from extended *in vitro* human blastocyst culture.^35^

During the first and second trimesters of pregnancy, the CTB, villous trophoblastic columns, and multinucleated intermediate-stage trophoblast cells strongly express GATA3 *in vivo,* while the STB are GATA3 negative.^70^ The villous CTB marker, TP63 (tumor protein 63), is essential for maintaining CTB stemness and is therefore only expressed in proliferative CTB.^71^ TP63 expression has also been shown to inhibit EVT migration.^72^ The continuous proliferation of CTB and continuous, but decreasing, differentiation of CTB into STB across the time course of culture under STB (suspension) conditions is likely reflected by the upregulation of TP63 transcripts and downregulation of SDC-1 transcripts (Figure 1C). In our study, EVT induction via exposure to a Matrigel substrate and EVT media resulted in decreased expression of TP63. GATA3 remained highly expressed in most of the EVT-like cells (Figures 4E, S2D, and S2E), which appear to penetrate the STB layer and migrate into the Matrigel (Figures 4A and 4F). Our findings align with the hypothesis that the column CTB ultimately give rise to the villous placenta.^35^ Additionally, we detected in our organoids other proliferative cells reported to be present in the first trimester human placenta, which are MKI67^+^TP63^−^ITGA2^+^, appear to retain their proliferative capacity^73^ and are thought to be a source of trophoblast progenitor cells located within the cell columns (Figures 4D and S2A).^36^^,^^73^

NOTCH1 has been detected in proliferative/mitotic proximal column trophoblasts and is hypothesized to define the EVT progenitor cell niche in early pregnancy, to facilitate invasive EVT lineage development and to support progenitor survival.^74^ NOTCH2 has been found in differentiated cells of the EVT lineage, and is thought to influence trophoblast migration.^75^ The presence of NOTCH1, NOTCH2, and EVT markers, such as HLA-G and hPL, within our EVT organoids suggests they accurately mimic the *in vivo* cell column of anchoring villi and its invasive EVTs (Figures 4D, 4F, S2C, and S2E). Additionally, the staining patterns in our trophoblast organoids likely represent the development of various trophoblast lineages over time within the culture conditions of both suspended and Matrigel-embedded cultures.

The key enzymes implicated in implantation, MMP-2 and MMP-9, play integral roles in the degradation of collagen I and IV, contributing to the remodeling of endometrial tissue throughout pregnancy.^76^ MMP-2 digests collagens I, II, and III, whereas MMP-9 is associated with the modulation of the biologically active form of vascular endothelial growth factor (VEGF), thereby exerting a crucial influence on the process of angiogenesis. Various regulators of MMP-9 expression have been explored, including hormones, extracellular matrix glycoproteins, as well as cytokines or growth factors.^77^ The absence of a maternal component of the maternal-fetal interface in our trophoblast organoid *in vitro* model may explain the absence of MMP-9 expression. Future, more complex co-culture models that include endometrial epithelia, endometrial stroma, and/or decidual immune cells may provide further insights into this finding.

In summary, we describe here a method to reliably generate human trophoblast organoids from human TS cells with a structure that more closely mimics the architecture of the human implanting embryo *in vivo* than prior trophoblast organoid models. Following the submission of our paper, two groups independently published their findings on trophoblast organoids derived from hTSCs.^45^^,^^78^ However, as far as we are aware, the trophoblast organoids surrounded by STB either were not demonstrated to differentiate into EVT cells^45^ or required forskolin treatment for full STB differentiation.^78^ Our model possesses the advantage of solely spontaneous STB differentiation conditions and EVT formation following STB establishment, mimicking the morphological environment of the human early placenta *in vivo*^79^ and enabling an implantation model that may not require the addition of exogenous hormones. Our *in vitro* model should improve our ability to study placental susceptibility to and vertical transmission of pathogens, maternal-fetal interactions across the villous placenta, and the effects of exposure to maternal blood-borne environmental toxins and pharmaceuticals in a physiologically relevant way.

### Limitations of the study

There are several limitations to this study. First, the apical-out model presented here was derived from hTSCs. Although we have replicated our results in four separate hTSC lines (two male and two female), we have not yet demonstrated a broader application of the method by using first trimester primary trophoblast cells or term trophoblast cells. Second, to date, the apical-out organoids have only been expanded and passaged a limited number of times in order to achieve optimized *in vitro* culture conditions. Third, the absence of extracellular matrix proteins during extended *in vitro* 3D culture may contribute to the relatively slow, but predictable, proliferation CTB cells in our cultures. Fourth, the organoids generated here comprise trophoblast derivatives, but not other fetal cells present within the placental villi *in vivo*, including fetal mesenchymal, immune and endothelial cells and maternal cells, including decidual cells and immune cells, that are normally found at the site of implantation. This limitation will be addressed in future experiments by using co-culture models. Fifth, like many TSC-derived models, the apical-out trophoblast organoids comprise several cell types and exhibit some degree of heterogeneity among individual organoids in term of size and number of cells. Transcriptome analysis is ongoing to better understand these organoids and the development of human placenta. Finally, some might argue that the presence of the coated bead in our culture system creates a round 2D culture condition, rather than a true 3D organoid, and that terms such as “organotypic cultures” or “self-organized 3D tissue” might be preferable. While the latter terms could be used to describe our system, we use the term organoids based on self-organization of these TSCs around the bead, the presence of multiple, differentiating cell layers that can replicate the complex interactions and *in vivo* structures of floating and anchoring villi, the reported use of modified 2D matrices in the creation of intestinal organoids^80^ and the historical use of the term “organoids” to refer to the culture of stem cell-based intestinal^81^ and lung^82^ organoids on beads.

## Resource availability

### Lead contact

Information and request for resources and reagents can be directed to and will be fulfilled by the lead contact, Danny J. Schust, MD at danny.schust@duke.edu.

### Materials availability

This study did not generate new or unique reagents. No new experimental animal models or cell lines were generated for this study or were research subjects enrolled.

### Data and code availability

• Data: All data supporting the findings of this study are included within the manuscript and supplemental information.
• Code: This study does not generate any original code.
• Additional Information: Any additional information required to reanalyze the data reported in this paper is available from the lead contact upon request.

## Acknowledgments

We appreciate Joshua Shelton and Shwetha Ramachandra from Dr. Bret D. Ulery’s group for their continuous support in modifying the polystyrene beads. We also sincerely thank the previous reviewers for their critical questions and valuable feedback, which have significantly contributed to enhancing the academic quality and completeness of our manuscript. This research is supported by grants 1R01HD094937 from the National Institutes of Health (D.J.S., T.E., L.C.S., and R.M.R.), R21AI145071 from the National Institutes of Health (D.J.S. and T.E.), and a Tier 2 grant from the University of Missouri (D.J.S., T.E., and L.C.S.).

## Author contributions

J.Z., T.E., and D.J.S. designed the study and were primarily responsible for experimental planning, data analysis, and manuscript writing. J.Z. contributed to experimental execution, including culturing the hTSC cell lines, RNA extraction, qPCR, immunofluorescence, and statistical analysis. M.A.S. contributed to data analysis and manuscript writing. K.J.D., M.M., and B.D.U. contributed to the polystyrene bead modification. Y.T. assisted with IHC and ELISA and provided technical support for data analysis. A.Z. assisted with DNA extraction and ELISA. T.P. assisted with ETM data analysis. All authors participated in data interpretation and manuscript revisions.

## Declaration of interests

The authors declare that the research was conducted in the absence of any commercial or financial relationships that could be construed as a potential conflict of interest.

## STAR★Methods

### Key resources table

**Table:** 

REAGENT or RESOURCE	SOURCE	IDENTIFIER
SDC-1	NOVUS	Cat# AF2780, RRID:AB_442186
KRT7	Dako	Cat# M7018, RRID:AB_2134589
hCGB	Abcam	Cat# Ab9582, RRID:AB_296507
GATA-3	Cell Signaling	Cat# 5852, RRID:AB_10835690
hCGA	NOVUS	Cat# NBP2-29428, RRID:AB_3276811
p63-α	Cell Signaling	Cat# 13109, RRID:AB_2637091
HLA-G	Santa Cruz	Cat# sc-21799, RRID:AB_627938
HLA-G (FITC)	NOVUS	NB110-55298F, RRID:AB_3177336
MKI67	Abcam	Cat# ab16667, RRID:AB_302459
ITGA2	Abcam	Cat# ab181548, RRID:AB_2847852
MMP2	Cell Signaling	Cat# 40994, RRID:AB_2799191
MMP9	Cell Signaling	Cat# 13667, RRID:AB_2798289
NOTCH1	Santa Cruz	Cat# sc-376403, RRID:AB_11149738
NOTCH2	Cell Signaling	Cat# 5732, RRID:AB_10693319
hPL	Sigma	Cat# 266A-15, RRID:AB_1159722
Alexa Fluor 647 Phalloidin	Invitrogen	Cat# A22287, RRID:AB_2620155
Alexa Flour 488 Donkey anti-Rabbit IgG	Invitrogen	Cat# A-21206, RRID:AB_2535792
Alexa Fluor 555 Donkey anti-Mouse IgG	Invitrogen	Cat# A-31570, RRID:AB_2536180
Alexa Fluor 647 Donkey anti-Goat IgG	Invitrogen	Cat# A-21447, RRID:AB_2535864

**Chemicals, peptides, and recombinant proteins**		

Sulfo-NHS	Thermo Scientific	PG82071
1-(3-dimethylaminoproyl)-3-ethylcarbodiimide hydrochloride	Thermo Scientific	Cat# 171440010
sodium bicarbonate	Sigma-Aldrich	Cat# S5761
ethylenediamine	Sigma-Aldrich	Cat# E26266
Corning® Collagen IV, mouse	Corning	Cat# 354233
Corning® Matrigel® Growth Factor Reduced GFR Basement Membrane Matrix	Corning	Cat# 356230
Corning® Cell Recovery Solution	Corning	Cat# 354253
Anti-Adherence Rinsing Solution	STEMCELL Technologies	Cat# 07010
Dimethyl Sulfoxide	Sigma-Aldrich	Cat# D8418
TrypLE Express Enzyme (1X), no phenol red	Gibco	Cat# 12604-021
Fetal Bovine Serum, embryonic stem cell-qualified, US origin	Gibco	Cat# 16141061
BSA (Fatty Acid Free)	Fisher Scientific	Cat# BP9704100
ITS-X	Gibco	Cat# 51500056
KnockOut Serum Replacement	Gibco	Cat# 10828010
hEGF (2D culture)	Sigma	Cat# E9644-.2MG
Stemolecule™ SB431542	Reprocell USA	Cat# 04-0010
Y27632	Reprocell USA	Cat# 04-0012-02
CHIR99021	Reprocell USA	Cat# 04-0004
A83-01	Reprocell USA	Cat# 04-0014
2-mercaptoethanol	Sigma	Cat# 21985-023
L-Ascorbic acid 2-phosphate sesquimagnesium salt hydrate	Sigma	Cat# A8960-5G
Valproic acid sodium salt	Sigma	Cat# P4543-10G
Human Neuregulin-1 (hNRG-1)	Cell Signaling	Cat# 26941S
Forskolin	Sigma	Cat# F6886-10MG
N2 supplement	Gibco	Cat# 17502048
B27 supplement minus vitamin A	Gibco	Cat# 12587010
N-acetyl-L-cysteine	Sigma	Cat# A9165-5G
Recombinant human EGF (3D culture)	PeproTech	Cat# AF-100-15
Recombinant human R-spondin 1	BioTechne	Cat# 4645-RS
GlutaMAX	Gibco	Cat# 35050061
Recombinant human FGF2	Peprotech	Cat# 100-18C
PGE2	Sigma	Cat# P0409
Recombinant human HGF	PeproTech	Cat# 100-39
donkey serum	Sigma	Cat# D9663
BSA (immunofluorescence)	Sigma	Cat# A7906
Triton X-100	Sigma	Cat# X100
DAPI	Invitrogen	Cat# D3571
VECTASHIELD Mounting Medium	Vector Labs	Cat# H-1200
formalin	Electron Microscopy Sciences	Cat# 15740-01
PFA	Electron Microscopy Sciences	Cat# 15710
osmium tetroxide	Ted Pella, Inc.	Cat# 18459

**Critical commercial assays**		

Vectastain Elite ABC-HRP kit	Vector Lab	PK-6100
hCG ELISA kits	Genway bio	Cat# GWB-BQK0F2
P4 ELISA kits	Genway bio	Cat# GWB-BQK0FC
PGF human ELISA kits	Invitrogen	Cat# EHPGF
RNeasy Mini kit	QIAGEN	Cat# 74104
cDNA synthesis kit	BIO-RAD	Cat# 1708890
SYBR Green PCR	MedChemExpress	Cat# HY-K0501
TURBO-DNA-free kit	Invitrogen	Cat# AM1907
Wizard® SV Genomic DNA Purification System	Promega	Cat# A2360

**Experimental models: Cell lines**		

hTSC CT27	hTSC CT27	hTSC CT27
hTSC CT29	hTSC CT29	hTSC CT29
hTSC BTS5	hTSC BTS5	hTSC BTS5
hTSC BTS11	hTSC BTS11	hTSC BTS11

**Software and algorithms**		

GraphPad Prism 9	GraphPad	www.graphpad.com/
LAS X	Leica	www.leica-microsystems.com/products/microscope-software/p/leica-las-x-ls/downloads/

**Other**		

polystyrene beads	Phosphorex	170

### Method details

#### Preparation of beads

Carboxylic acid-functionalized polystyrene beads (d=200 μm, -COOH) were conjugated with -NH2 via EDC-NHS coupling using a commercial protocol.^83^ In brief, 10 mg of 200 μm polystyrene beads (-COOH) were suspended in 10 mL of double-distilled water (ddH2O) (10 mg/mL). Sulfo-NHS and 1-(3-dimethylaminoproyl)-3-ethylcarbodiimide hydrochloride (EDC-HCl) were sequentially added to a final concentration of 0.25 mg/mL to the bead suspension and shaken for 20 min at room temperature. Then, sodium bicarbonate (NaHCO3, 50 mM, PH=8) and ethylenediamine (5 mg/mL) were added to the bead suspension and shaken at room temperature for 2 h. After the conjugation reaction, the beads were sterilized by three 15-minute centrifuge cycles with fresh 70% ethanol and washed with sterile PBS. Prior to use, the modified polystyrene beads (-NH2) were coated with 5 μg/ml Collagen IV at 37°C for at least 2 h.

#### Human TSC culture and trophoblast organoid differentiation

The hTSC lines, CT27, CT29, BTS5, and BTS11 were derived from first-trimester placentas (CT) or blastocysts (BT) by Okae et al.^15^ and kindly provided by Dr. Michael Soares (UMKC) and Dr. Liping Feng (Duke University) and were cultured in hTSC 2D medium [DMEM/F12 supplemented with 0.1mM 2-mercaptoethanol, 0.2% FBS, 0.3% bovine serum albumin (BSA), 1% I=insulin-transferrin-selenium-ethanolamine supplement (ITS-X), 1.5 mg/ml L-ascorbic acid, 50 ng/ml hEGF, 2mM CHIR99021, 0.5mM A83-01, 1mM SB431542, 0.8 mM valproic acid and 5 mM Y27632]. Cells were dissociated into single cells by TrypLE, washed with DMEM/F12 culture medium and pelleted. The cell pellet was resuspended in hTSC 2D medium and 5X104 cells were incubated with 0.5 mg collagen IV pre-coated beads (approximately 40 beads) in 3 ml hTSC 2D medium supplemented with 5μM Y27623 in a round-bottom test tube at 37°C for 4 h, the tube was stirred gently for 1 minute every 30 minutes. Cells were then transferred to Ultra-Low attachment 6-well plates and cultured for additional 48 h. The surfaces of the collagen-coated beads were largely covered with cells within 24 h. On day 3, the medium was changed to trophoblast organoid medium (TOM)16 [DMEM/F12, 1X N2 supplement, 1X B27 supplement minus vitamin A, 1.25 mM N-acetyl-L-cysteine, 1% GlutaMAX, (TOM basal medium)], supplemented with 500 nM A83-01 and 1.5mM CHIR99021 to induce trophoblast organoid formation. On day 5, the culture medium was changed to TOM2 (TOM basal medium supplemented with 500 nM A83-01, 1.5mM CHIR99021, 80 ng/ml human R-spondin1, 50 ng/ml hEGF, 100 ng/ml hFGF2, 50 ng/ml hHGF, 2mM Y-27632). The medium was replaced every 3-4 days until day 10.

#### Derivation of hTSC-derived matrigel-embedded trophoblast organoids

hTSCs were dissociated into single-cells with TrypLE and washed twice in Advanced DMEM/F12. Five thousand cells were suspended in 60 μL Matrigel droplets and seeded in 12-well plates, followed by polymerization of the Matrigel droplets at 37°C for 30 minutes. Plates were subsequently removed from the incubator and 1.5 ml of TOM was added. 1.5 ml of TOM was changed every other day and organoids were maintained for 10 days.

#### Generation of EVT-like cells from trophoblast organoids

On day 7 of differentiation, 4-5 STB trophoblast organoids, as generated above, were transferred to an Eppendorf tube with a 200-μL wide bore pipette tip. The organoids were resuspended in 20 μL Matrigel. The drop was the placed in either 34-mm TPP dishes or MatTek Corporation dishes. Matrigel was allowed to gel at 37°C for 15 min, and the drop then overlaid with 3ml EVT differentiation medium^16^ (EVTM: advanced DMEM/F12, 0.1 mM 2-mercaptoethanol, 0.3% BSA, 1% ITS-X supplement, 100 ng ml−1 NRG1, 7.5 μM A83-01 and 4 % knockout serum replacement) under an atmosphere of 5% (v/v) CO2/air at 37°C. The medium was changed on day 11 (4 days after Matrigel embedding) and organoids collected on day 14.

#### Whole mount immunofluorescence staining and confocal microscopy

Organoids were fixed with 4% (v/v) paraformaldehyde (PFA) in PBS for 30 min and permeabilized in 0.4% Triton X-100/PBS for 30 min. Organoids were placed in 10% (v/v) donkey serum/5% (w/v) BSA /0.1% Triton X-100/ in PBS as a blocking reagent for 2 h. Cells were then incubated with primary antibodies at 4°C for 48 h. DAPI and secondary antibody staining was performed with either Alexa Fluor 555-, 647-, or 488-labeled detection reagents at a 1:300 dilution at 4°C overnight. Samples were then mounted in mounting media and images captured under a Leica TCS SP8 confocal laser scanning microscope. For live cell imaging, organoids were grown in 35mm dishes with coverslips and, just prior to imaging, incubated for 1h at 37°C in FITC labeled HLA-G antibody at 1:100. Organoids were then washed twice in DMEM/F12, transferred to EVTM and immediately imaged under a Leica TCS SP8 confocal laser scanning microscope.

#### H&E staining and immunohistochemistry

Organoids were fixed in 10 % (v/v) neutral buffered formalin. Paraffin-embedded organoids were sectioned and then stained with hematoxylin-eosin (H&E) or processed for immunofluorescence staining and immunohistochemistry. For immunofluorescence staining, slides were placed in boiling citric acid buffer (pH 6.0), non-specific antigens blocked in 5% (v/v) donkey serum/5 % (w/v) BSA in PBS for 1 h, and incubated with primary antibodies at 4°C overnight. The sections were then incubated with fluorescent-conjugated secondary antibodies for 1 h at room temperature. Images were captured under a Zeiss Axiovert 200M with a Leica DFC290 color camera. For immunohistochemistry, sections were rehydrated through a xylene substitute and graded alcohol series. Antigen retrieval was performed as previously described.^84^ Immunoperoxidase staining was performed by using a Vectastain Elite ABC-HRP kit, and images were captured with a Leica DM 5500B upright microscope with a color digital camera.

#### Transmission electron microscopy

The organoids were fixed in 0.1% (w/v) glutaraldehyde/4 % formaldehyde (v/v) in 0.1 M sodium cacodylate buffer pH 7.2 at room temperature for 30 min and post-fixed with 1 % (w/v) osmium tetroxide. Specimens were dehydrated through a graded ethanol series, placed in acetone and infiltrated with Epon by means of a Pelco Microwave (Ted Pella, CA). Thin sections (85 nm) were mounted on formvar/carbon-coated slot grids and post-stained with 2 % uranyl acetate and Sato’s triple lead stain. Sections were examined on a JEOL 1400 transmission electron microscope at 80kV.

#### Scanning electron microscopy

Samples were collected and processed for scanning electron microscopy (SEM). Unless otherwise stated, all reagents were purchased from Electron Microscopy Sciences and all specimen preparation performed at the Electron Microscopy Core Facility, University of Missouri. The samples were fixed in 2 % paraformaldehyde, 2 % glutaraldehyde in 100 mM sodium cacodylate buffer pH 7.35. Fixed organoids were plated overnight on coverslips pre-coated with medium to ensure adhesion and rinsed with 100 mM sodium cacodylate buffer, pH 7.35 containing 130 mM sucrose. Secondary fixation was performed in 1 % osmium tetroxide in cacodylate buffer by using a Pelco Biowave (Ted Pella, Inc.) operated at 100 Watts for 1 min. Specimens were next incubated at 4°C for 1 h, then rinsed with cacodylate buffer and finally with distilled water. A graded dehydration series (per exchange, 100 Watts for 40 s) was performed with ethanol in the Pelco Biowave. Samples were dried by using the Tousimis Autosamdri 815 (Tousimis, Rockville, MD) and samples were sputter coated with 5nm of platinum using the EMS 150T-ES Sputter Coater. Images were acquired with a FEI Quanta 600F scanning electron microscope (FEI, Hillsboro, OR).

#### Immunoassays

To assess the time course of hormone production, organoid culture medium was collected on days 1, 3, 5 8, and 10. Human CG (hCG; human chorionic gonadotrophin), progesterone (P4) and PGF (placental growth factor) concentrations were measured with solid-phase sandwich hCG ELISA kits, P4 ELISA kits and PGF human ELISA kits, respectively, by following the manufacturer recommended protocols. Samples were collected from three independent experiments for each time point and were run in duplicate. To compare the hormone production between Matrigel-embedded organoids and proper-polarized organoids, the suspended organoids were transferred to a 12-well plate treated with Anti-Adherence Rinsing Solution, and 1.5 ml freshly prepared TOM medium was added Simultaneously, the media of Matrigel embedded organoids were replaced with 1.5 ml TOM.

Culture medium collected after a 24-hour period was used for ELISA analysis. Matrigel-embedded organoids were dissociated from Matrigel droplets by incubating them in Cell Recovery solution on ice for 40 minutes. Subsequently, whole organoids were harvested, and total DNA was isolated using Promega Wizard® SV Genomic DNA Purification System in order to normalize immunoassay results to cell density levels. DNA quality and concentration were determined in a Nanodrop ND-1000 Spectrophotometer. Total DNA was used for normalization.

#### Real-time qPCR

To remove the few small contaminating cell aggregates that differed from the vast majority of coated beads, organoids containing beads were selected under a ZEISS Stemi 508 stereo microscope for RNA extraction. Total RNA was extracted with a RNeasy Mini kit, and RNA treated with a TURBO-DNA-free kit to remove genomic DNA. RNA was reverse-transcribed with a cDNA synthesis kit. Primers were synthesized by Integrated DNA Technologies (IDT). Primer sequences are provided in qPCR Primer table. Real-time qPCR was performed with SYBR Green PCR on a CFX Connect Real-Time PCR System (BIO-RAD). GAPDH was used as the endogenous control (reference gene). Thermal cycling conditions were as follows: 95°C for 5 min, followed by 45 cycles of: denaturation at 95°C for 10 sec, annealing at 56°C for 10 sec and extension at 72°C for 10 sec. CT values were normalized to the endogenous control (GAPDH) and fold-change values calculated relative to hTSC grown in 2D by the 2−ΔΔCT procedure described elsewhere.^85^ results were reported as relative expression levels compared to the individual control sample in each assay.

**Table:** qPCR Primer Table

Gene	Primer sequence
*TP63*^15^	AGAAACGAAGATCCCCAGATGA
	CTGTTGCTGTTGCCTGTACGTT
*MIK67*^86^	GAAGAGCTCCTAGCAGTCG
	GGCCACTTCTTCATTCCAG
*TEAD4*^15^	CAGGTGGTGGAGAAAGTTGAGA
	GTGCTTGAGCTTGTGGATGAAG
*KRT7*^87^	AAGAACCAGCGTGCCAAG T
	TCCAGCTCCTCCTGCTTG
*GATA3*^1^	TACGTGCCCGAGTACAGCTT
	ACTCCCTGCCTTCTGTGCT
*CGB*^1^	GTCAACACCACCACCATGTGTGC
	GGTAGTTGCACACCACCTGA
*SDC-1*^15^	CTATTCCCACGTCTCCAGAACC
	GGACTACAGCCTCTCCCTCCTT
*GAPDH*^1^	CTGGGGCTGGCATTGCCCTC
	GGCAGGGACTCCCCAGCAGT

### Quantification and statistical analysis

Statistical analyses were performed using GraphPad Prism 9 software. For qPCR and immunoassays, data analyzed over time were subjected to two-way ANOVA, followed by the Bonferroni test for pairwise comparisons. An unpaired t-test was used to compare immunoassay data between two different organoid groups established using distinct methods. Values of *P* < 0.05 were considered statistically significant.
