# Supplementary material for: Development of apical out trophoblast stem cell derived organoids to model early human pregnancy
Source: iScience. 2025 Feb 25;28(3):112099. doi: 10.1016/j.isci.2025.112099 (PMC11930733; doi:10.1016/j.isci.2025.112099)
Supplement: Document S1. Figures S1 and S2 [file mmc1.pdf]

iScience, Volume *28*

Supplemental information

Development of apical out trophoblast stem cell derived organoids to model early human pregnancy

J. Zhou, M.A. Sheridan, Y. Tian, K.J. Dahlgren, M. Messler, T. Peng, A. Zhao, T. Ezashi, L.C. Schulz, B.D. Ulery, R.M. Roberts, and D.J. Schust

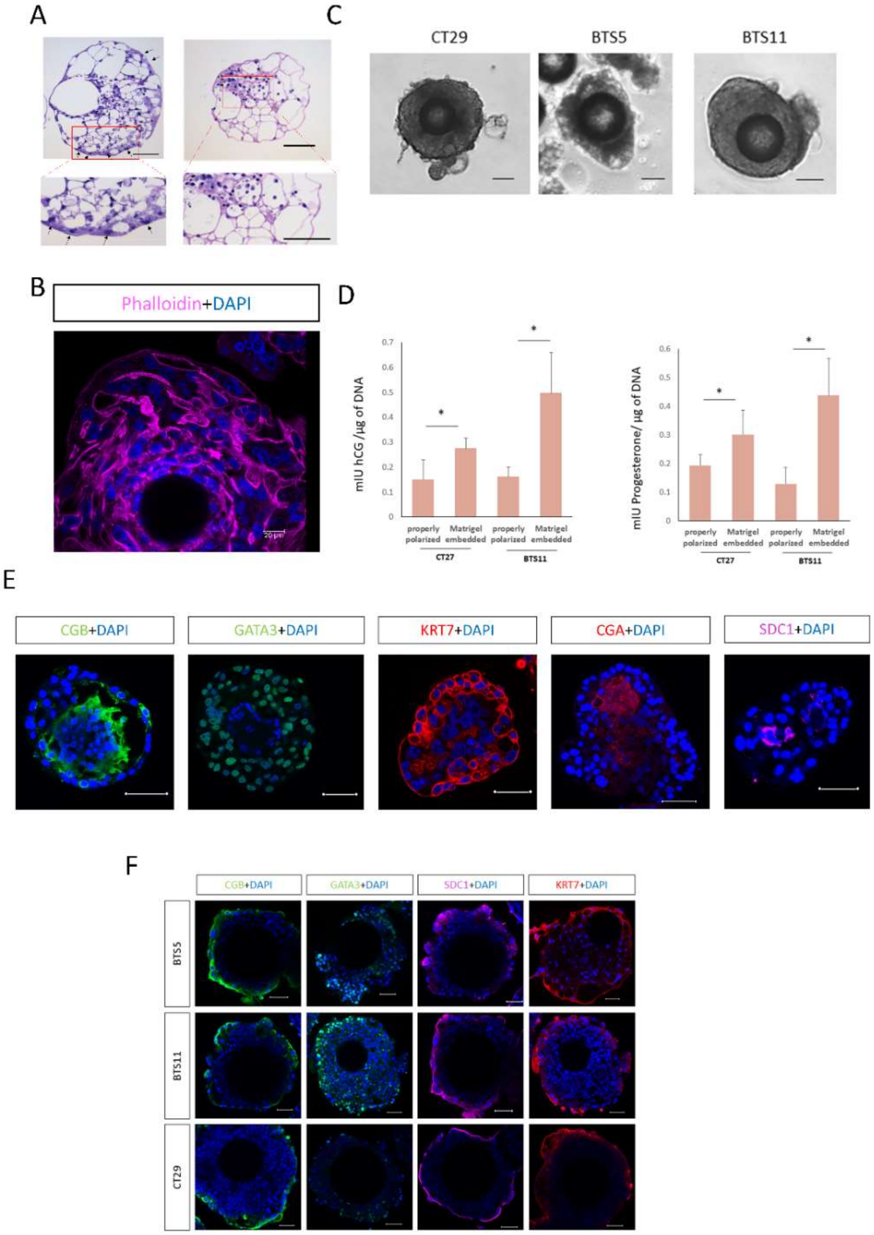

Figure S1: Comparison of new apical-out trophoblast organoids and standard apical-in trophoblast organoids. A. Hematoxylin and eosin (H&E) staining of organoids on day 10 revealed multinucleated areas (black arrows) that remain inside the organoid in absence of a bead. (left panel: with beads; right panel: without beads) Scale bars, 50µm. B. Immunostaining with phalloidin in apical-out, CT27-derived organoids on D10. Scale bar, 20 µm. (n=3) C. Bright-field images of CT29, BTS5 and BTS11-derived trophoblast organoids. Scale bars, 200µm. D. Daily (24 hr) hCG and progesterone production was assessed using ELISA on D10 of apical-out suspended and matrigel-embedded organoids derived from CT27 and BTS11. Protein concentrations by ELISA were normalized to the DNA content of each culture. Values are means ± SEs for three individual experiments. An unpaired t-test was used to compare immunoassay data between two different organoid groups, *$P < 0.05$. (n=3) E. Immunostaining for CGB, GATA3, KRT7, CGA and SDC-1 in matrigel-embedded organoids (n=2) F. Immunostaining of CGB, GATA3, KRT7 and SDC-1 in apical-out organoids derived from BTS5, BTS11 and CT29 on D10. Scale bars, 50µm. *$P < 0.05$. (n=2)

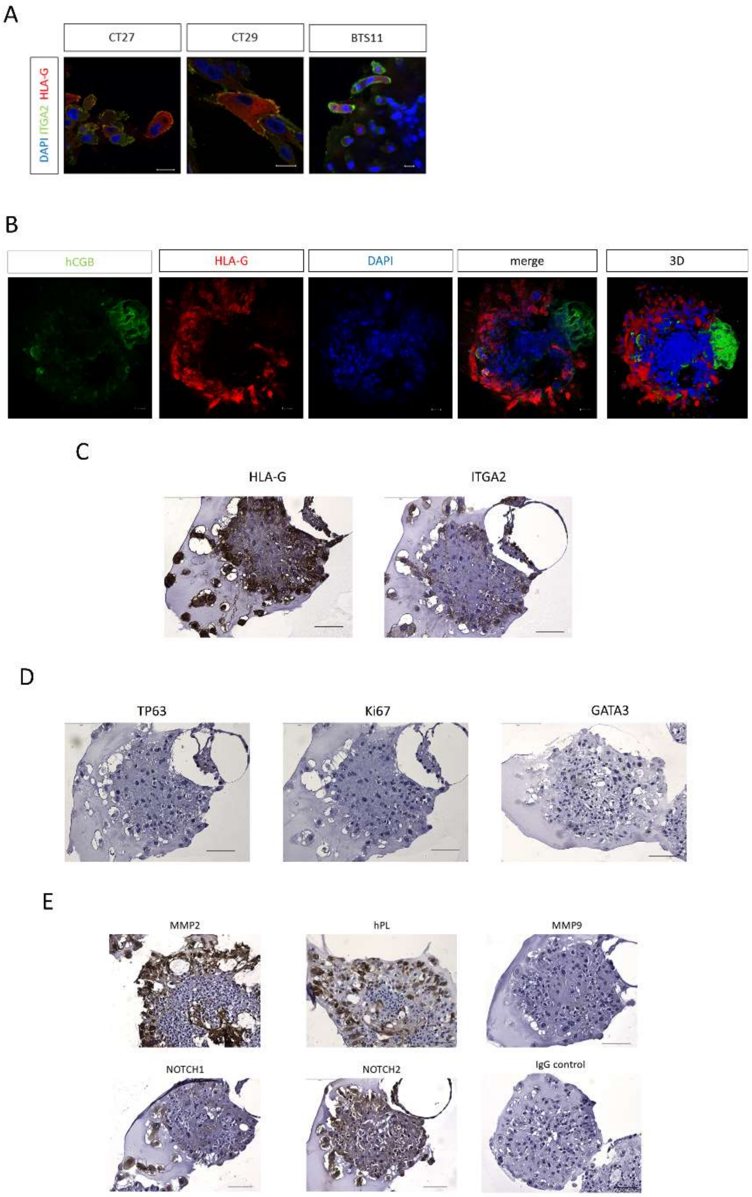

Figure S2 EVT trophoblast organoids express markers of infiltration, invasion, and proliferation. A-B. On Day 14, organoids in EVTM were co-stained for ITGA2 and HLA-G (A) or HLA-G and hCGB (B). ITGA2 and HLA-G are co-expressed on both migrating and invading cells. hCGB is expressed mainly in cells positioned along the external surface of the organoids, while the migrating and invading cells are HLA-G positive. Scale bars, 50 µm. (n=2) C-E. Day 14 paraffin-embedded sections of BTS11 organoids grown in EVTM for 7 days, stained for the EVT markers, HLA-G and ITGA2 (C); the CTB markers TP63, MKI67 and GATA3 (D), the infiltrating trophoblastic markers, MMP2, MMP9, hPL and the column trophoblastic markers, NOTCH1 and NOTCH2 (E). Scale bars, 100 µm.
